# Comprehensive elaboration of the cGAS-STING signaling axis in cancer development and immunotherapy

**DOI:** 10.1186/s12943-020-01250-1

**Published:** 2020-08-27

**Authors:** Juyan Zheng, Junluan Mo, Tao Zhu, Wei Zhuo, Yueneng Yi, Shuo Hu, Jiye Yin, Wei Zhang, Honghao Zhou, Zhaoqian Liu

**Affiliations:** 1 Department of Clinical Pharmacology, Hunan Key Laboratory of Pharmacogenetics, and National Clinical Research Center for Geriatric Disorders, Xiangya Hospital, Central South University, Changsha, 410008 People’s Republic of China; 2 Institute of Clinical Pharmacology, Engineering Research Center for applied Technology of Pharmacogenomics of Ministry of Education, Central South University, Changsha, 410078 People’s Republic of China; 3 Shenzhen center for chronic disease control and Prevention, Shenzhen, 518020 People’s Republic of China; 4 Hunan Yineng Biological Medicine Co., Ltd, Changsha, 410205 People’s Republic of China; 5 Department of Nuclear Medicine, Key Laboratory of Biological Nanotechnology of National Health Commission, Xiangya Hospital, Central South University, Changsha, Hunan 410008 People’s Republic of China

**Keywords:** cGAS-STING, Innate immunity, Type I interferon, STING agonists, Antitumor response, Cancer development

## Abstract

Cellular recognition of microbial DNA is an evolutionarily conserved mechanism by which the innate immune system detects pathogens. Cyclic GMP-AMP synthase (cGAS) and its downstream effector, stimulator of interferon genes (STING), are involved in mediating fundamental innate antimicrobial immunity by promoting the release of type I interferons (IFNs) and other inflammatory cytokines. Accumulating evidence suggests that the activation of the cGAS-STING axis is critical for antitumor immunity. The downstream cytokines regulated by cGAS-STING, especially type I IFNs, serve as bridges connecting innate immunity with adaptive immunity. Accordingly, a growing number of studies have focused on the synthesis and screening of STING pathway agonists. However, chronic STING activation may lead to a protumor phenotype in certain malignancies. Hence, the cGAS-STING signaling pathway must be orchestrated properly when STING agonists are used alone or in combination. In this review, we discuss the dichotomous roles of the cGAS-STING pathway in tumor development and the latest advances in the use of STING agonists.

## Introduction

The discovery of phagocytosis in 1883 advanced the understanding of innate immunity, the first line of host defenses against infection by various pathogens [1]. Protection against infection depends on pattern-recognition receptors (PRRs), which recognize microbial products, coordinate antimicrobial defenses and activate adaptive immunity [2]. Abnormal RNA or DNA, RNA-DNA hybridization and cyclic dinucleotides derived from microbes are usually considered pathogen-associated molecular patterns (PAMPs) [2, 3]. Cells associated with innate immunity recognize different microbial PAMPs through specific PRRs, thereby playing key roles in host resistance to microbial infection [4]. The pathways governing RNA recognition, such as retinoid acid inducible gene I (RIG-I)-like receptors, have been reviewed elsewhere and will not be covered herein. In the case of DNA recognition, one of the best known PRRs is Toll-like receptor 9 (TLR9), which senses extracellular CpG hypomethylated DNA that has entered the cytosol through the phagosome-lysosome system [5]. In addition, the AIM2-like receptor (AIM2) inflammasome can be triggered after the entry of double-stranded DNA (dsDNA) into the cytosolic compartment, which induces the proteolytic maturation of proinflammatory cytokines (such as IL-1β and IL-18) and the activation of gasdermin D, leading to pyroptosis [6–9]. Nevertheless, the most notable PRR is cGAS, a direct cytosolic dsDNA sensor, which was identified by Dr. Chen’s group in 2013 [10]. Once cGAS binds to dsDNA, the cGAS-STING pathway is activated to further induce the expression of type I IFNs and other inflammatory cytokines, thus triggering innate immune responses [11]. Mounting evidence suggests that cGAS-STING signaling not only plays pivotal roles in the host defense against microbial infection but also modulates tumorigenesis. Hence, in this review, we summarize the mechanism of cGAS-STING activation and elaborate findings regarding its dual effects on tumor development. Current advances in the use of STING agonists as a novel strategy for antitumor therapy are also reviewed.

## Insights into the cGAS-STING signal transduction cascade

**Fig. 1:**
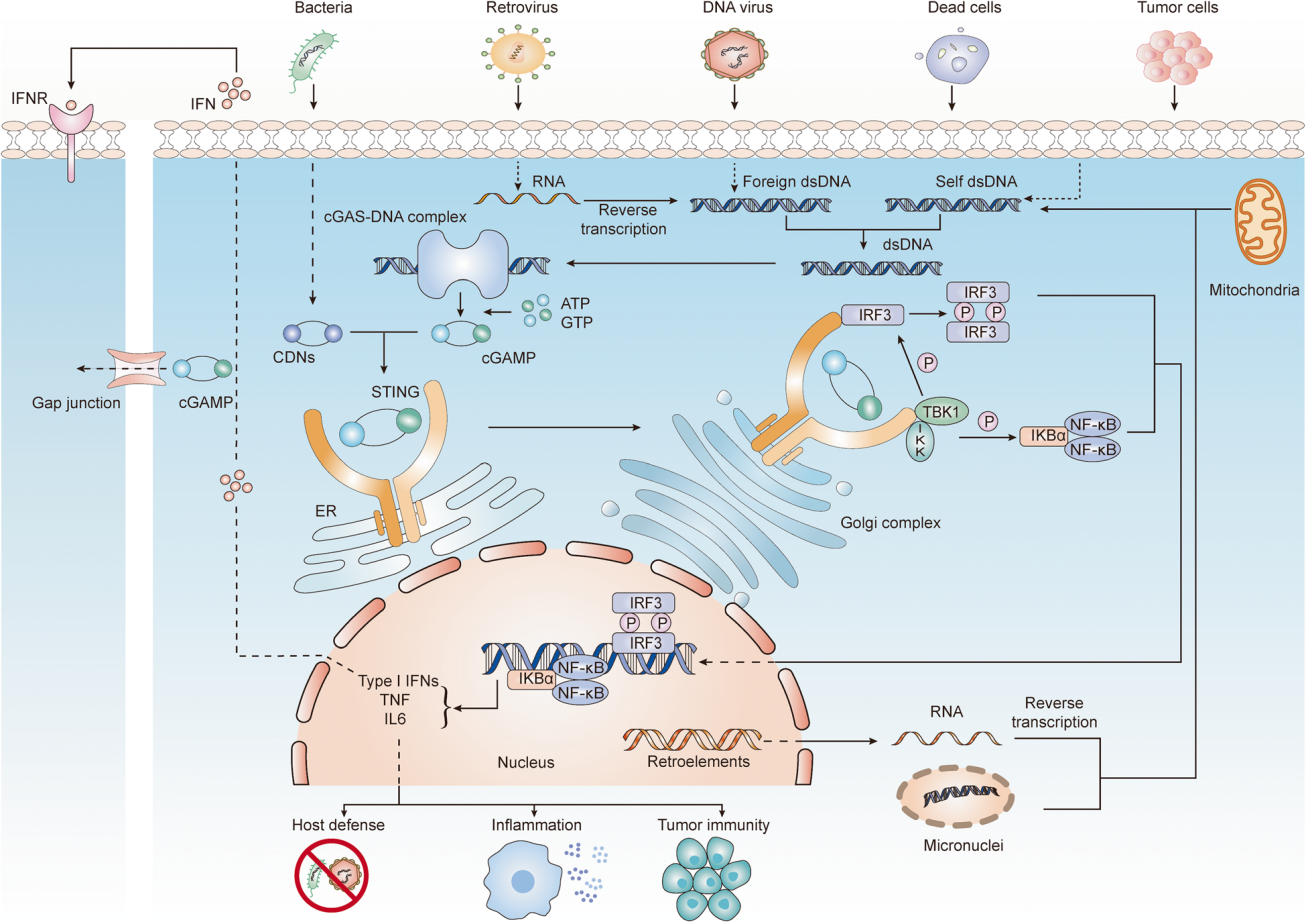
The cGAS-STING DNA sensing signaling pathway. Various DNA derived from virus, dying tumor cells or nucleus and mitochondria binds to and activates the cytosolic DNA sensor cGAS, cGAS catalyzes the synthesis of 2′3′-cGAMP in the presence of ATP and GTP, then 2′3′-cGAMP binds to the ER adaptor STING, which also can be activated by CDNs derived from bacteria. Upon activation, STING translocates from ER to Golgi compartments, where it activates TBK1 and IKK, which phosphorylate IRF3 and IκBα respectively. Then IRF3 and IκBα dimerize and enter nucleus, initiating the transcription of Type I IFN, TNF and IL6. The primary roles of these cytokines are reflected in host defense, inflammation and antitumor immunity

cGAS is an innate immune sensor that identifies various cytosolic dsDNA, including DNA with viral, bacterial, mitochondrial, micronuclei, and retroelement origins, which can be mainly divided into pathogen-derived DNA and self-DNA (Table 1). In the cytoplasm, cGAS is activated by interacting with dsDNA in a sequence-independent but length-dependent manner [32–34]. Structural and biochemical analyses have revealed that the C-terminal lobe of cGAS contains a conserved zinc-ion-binding module that mediates DNA binding and cGAS dimerization [35, 36]. DNA ligands promote cGAS activation primarily by inducing conformational changes around the catalytic site, and in the DNA-binding structures of cGAS, the GS-containing loop undergoes conformational change to maintain stability, which is a major mechanism of cGAS activation by DNA [37]. In addition to the primary DNA-binding site mentioned above, the secondary site located beside the primary site is a helix formed between strands β7-β8 and several surface-exposed loops [38]. The proximity of the two DNA-binding sites in cGAS leads to a 2:2 cGAS:DNA complex assembly, in which two cGAS molecules embrace two molecules of dsDNA [39, 40]. The cGAS dimers are organized in “head-to-head” alignment next to the DNA [41] and thus form stable “ladder-like” networks between one long curved dsDNA helix or two independent dsDNA strands [33, 42]. In this way, each individual cGAS-dsDNA complex can be cooperatively stabilized and can lead to stronger enzymatic activity, which may provide a possible explanation for longer dsDNA as more likely to activate cGAS [43]. In addition, long DNA is more efficient than short DNA in driving the liquid-liquid phase separation of cGAS, and the formation of cGAS liquid-like droplets is critically dependent on the concentration of cGAS and DNA in the cytoplasm [44]. cGAS and dsDNA are spatially concentrated in liquid droplets to facilitate cGAS dimerization and activation [45–47]. Once cGAS and dsDNA interacts, structural switches rearrange the catalytic pocket to enable cGAS to catalyze the synthesis of 2′3′-cyclic GMP-AMP (2′3′-cGAMP), with ATP and GTP as substrates. The first step in this process is the formation of a linear dinucleotide 5′-pppG (2′-5′)pA with ATP serving as the donor and 2′-OH on GTP serving as the acceptor. Then, the intermediate product flips over in the catalytic pocket, placing GTP at the donor position and AMP at the acceptor position to form a second 3′-5′ phosphodiester bond [32, 35, 48]. Notably, although dsRNA or single-strand DNA (ssDNA) is able to bind to cGAS, neither can rearrange the catalytic pocket, which may explain the exclusive activation of cGAS by dsDNA. Ultimately, cGAMP acts as a second messenger to bind to and activate STING, a small endoplasmic reticulum (ER)-located protein (~ 40 KD) with four putative transmembrane domains [49, 50]. Normally, in a resting state, STING is retained in the ER by interacting with the Ca^2+^ sensor stromal interaction molecule 1 (STIM1) [51]. The cytosolic ligand-binding domain (LBD) of STING exists as the most functional unit capable of integrating with 2′3′- cGAMP or CDNs (cyclic dinucleotides) such as c-di-AMP, c-di-GMP or 3′3′-cGAMP from bacteria. Upon interaction, the obvious closure of the ligand binding pocket in the LBD is observed, which is related to the activation of STING [52]. Next, STING transforms into a tetramer through a high-order oligomerization reaction and is translocated from the ER to the perinuclear area facilitated by cytoplasmic coat protein complex II (COPII) and ADP-ribosylation factor (ARF) GTPases [53, 54]. In the Golgi, STING is palmitoylated at two cysteine residues (Cys88 and Cys91), a posttranslational modification necessary for STING activation [55]. Modified STING recruits the kinase TANK-binding kinase 1 (TBK1); in turn, the C-terminal domains of STING are phosphorylated by TBK1; and then, phosphorylated STING recruits interferon regulatory factor 3 (IRF3), which is also phosphorylated by TBK1 and dimerizes; ultimately, dimerized IRF3 enters the nucleus and exerts its function in the transcription of type I IFNs and interferon-stimulated genes (ISGs) [56]. In parallel, STING can also bind to and stimulate IκB kinase (IKK) to mediate the production of nuclear factor-κB (NF-κB)-driven inflammatory genes. Upon signal transduction termination, STING is transferred to endolysosomes for degradation [14]. Considering that cGAMP can be transferred through gap junctions or delivered in viral/exosome packages, cGAS-STING signaling may be activated in the cytoplasm without dsDNA [57, 58]. Moreover, newly produced type I IFNs activate heterodimer interferon receptors (IFNAR1 and IFNAR2) through paracrine signaling and thus induce the transcription of ISGs [59, 60]. In summary, once virus-derived DNA and self-DNA are located in the cytoplasm, they can be sensed by cGAS, and a 2:2 cGAS:dsDNA complex is formed to catalyze the synthesis of 2′3′-cGAMP with ATP and GTP. Then, 2′3′-cGAMP and bacteria-derived CDNs induce STING activation and mediate the release of downstream type I IFNs, TNF-α and IL-6, which are prerequisites for antimicrobial defense and antitumor effects. The whole process shows that the dsDNA-cGAS-STING axis can lead to the activation of both innate and adaptive immunity (Fig. 1).

**Table 1:** Classification of the cytosolic dsDNA that activates the cGAS-STING signaling axis

Classification	Source of dsDNA		Possible mechanisms	References
Self-DNA	Micronuclei		Rupture of the micronuclei membrane leads to exposure of chromatin DNA that is recognized by cGAS, which activates the cGAS-STING pathway.	[12]
	Mitochondrion		Mitochondrial stress induces mtDNA leakage into the cytosol, thus activating the STING pathway and inducing production of cytokines.	[13]
	Nuclear RNA		Facilitated by endogenous retroelements, nuclear RNA can be reversely transcribed into DNA that activates cGAS-STING signaling.	[10]
Pathogen-derived DNA	DNA virus	HSV1, HSV2, KSHV, adenovirus, vaccinia virus, cytomegalovirus, papillomavirus, murine gamma-herpesvirus 68	DNA viruses invade host cells and release pathogen-derived DNA to induce STING activation.	[14–20]
	Retrovirus	HIV, SIV, murine leukemia virus	DNA intermediates generated from reverse transcription may be recognized by cGAS to stimulate downstream STING signaling.	[11]
	RNA virus	West Nile virus, dengue virus, VSV, SARS-COV-2	Infection with RNA viruses might cause cellular damage and cell death, which results in the release of cellular DNA and further activation of the cGAS-STING axis; SARS-CoV-2 binding to ACE2 can lead to excessive angiotensin II signaling that activates the STING pathway in mice.	[21–23]
	Bacteria	*Listeria monocytogenes*, *Mycobacterium tuberculosis*, *Listeria*, *Shigella*, *Francisella*, *Chlamydia* and *Neisseria*	Bacteria produce CDNs, such as cyclic di-GMP and cyclic di-AMP, which can directly bind to and activate STING.	[10, 24–31]

*HSV1,* herpes simplex virus 1; *HSV2,* herpes simplex virus 2; *KSHV,* Kaposi sarcoma–associated herpesvirus; *HIV,* human immunodeficiency virus; *SIV,* simian immunodeficiency virus; *VSV,* vesicular stomatitis virus; *CDNs,* cyclic dinucleotides; and *SARS-COV-2,* severe acute respiratory syndrome coronavirus 2

## The antitumor functions of the cGAS-STING signaling pathway

Recent evidence has revealed the close association of the cGAS-STING pathway with cancer development. This signaling pathway is generally regarded as a potent regulator of cancer immunity. A STING-mediated immune supportive microenvironment can hamper malignancy occurrence [61].

### Tumor cell cytosolic dsDNA induces STING activation

Under normal circumstances, DNA is strictly unaffiliated with the cytoplasm in eukaryotic cells to avoid autoimmunity [62]. However, DNA leaks aberrantly in tumor cells [63, 64]. Cancer cells share common features, including genome instability, tumor suppressor gene mutation or deletion, oxidative stress and vigorous metabolism [65]. Under these intense states, nuclear and mitochondrial DNA is fragile and easily damaged, which leads to eventual DNA leakage in the forms of micronuclei, chromatin fragments and/or free telomeric DNA [12, 66, 67]. Chromosomal instability (CIN) is the primary source of cytoplasmic DNA in malignant cells and is generally associated with tumor progression, distant metastasis and therapeutic tolerance [61]. Excessive proliferation of cancer cells results in unstable genomes [68, 69], usually characterized by chromosomal mis-segregation during mitosis. Due to defects in segregation, lagging chromosomes generate micronuclei in a cell cycle-dependent manner [12]. The vulnerable membrane of micronuclei easily exposes the inner DNA to the cytoplasm and activates the cGAS-STING signaling axis [12, 70]. Exogenous stimuli such as chemotherapy and irradiation can also cause DNA damage. In addition to leaked nuclear DNA, oxidative stress-induced mitochondrial DNA leakage is another crucial initiator of STING pathway activation. Several anticancer treatments that precisely attack mitochondrial membranes result in efflux and cell death. Therefore, the permeabilization of mitochondria membranes provides a reasonable explanation for mitochondrial DNA escape [71, 72]. Other sources, such as apoptotic cell-derived DNA, exosomal DNA (ExoDNA), and transposable elements, have also been demonstrated to evoke cGAS–STING activation in tumor cells [73, 74].

### Type I IFNs: mediators of STING and adaptive antitumor effects

cGAS-STING signaling exerts antitumor functions in cancer cells both in an autonomous and nonautonomous manner. On the one hand, DNA damage can provoke acute STING signal transduction and induce cellular senescence, an irreversible cell cycle arrest state, which thwarts the aberrant proliferation of tumor cells through acquisition of the senescence-associated secretory phenotype (SASP), which is associated with the release of abundant inflammatory mediators, proteases and growth factors [41, 75, 76]. In contrast to undergoing senescence, tumor cells also directly propel apoptosis processes by upregulating proapoptosis protein BCL2-associated X (BAX) and downregulating the BCL2 apoptosis regulator [77]. On the other hand, STING activation in tumor cells not only facilitates the transcription of downstream type I IFNs to induce dendritic cell maturation but also recruits supportive immune cells for direct, nonspontaneous tumor elimination [78].

STING activation in nonmalignant cells causes tumor suppressive effects as well. STING signaling protects against colitis-associated carcinomas (CACs) induced by azoxymethane (AOM) and dextran sulfate sodium (DSS), which induce DNA damage in intestinal epithelial cells and further trigger STING activation. Downstream cytokines of STING signaling, such as IL-1β and IL-18, prevent neoplastic transformation by facilitating wound repair. More importantly, STING signaling can also provoke cytotoxic T cell responses to control tumorigenesis [66]. Necrotic cancer cells are commonly engulfed by antigen-presenting cells, especially the basic leucine zipper transcription factor ATF-like 3 (BATF3)-driven lineage of dendritic cells (DCs) [59]. BATF3 DCs take in tumor-associated antigens and migrate towards the tumor-draining lymph node via the lymphatic system, where they cross-prime tumor-specific CD8^+^ T cells. Then, CD8^+^ T cells undergo activation and clonal expansion in the lymph nodes and are trafficked through blood vessels to kill tumor cells. In turn, damaged cancer cells release more antigens that are further captured by DCs, the whole process forms a positive feedback loop called the cancer-immunity cycle [79]. Tumor eradication can be achieved by multiple processes in the cancer-immunity cycle, including tumor antigen capture and presentation and T cell priming and activation, with tumor antigen-specific T cell priming and activation relying on DCs and type I IFN release [80]. The involvement of type I IFNs in innate immune sensing and adaptive immunity provides a reasonable hypothesis for exploring candidate PRR pathways as potential immunomodulators. Mice lacking TLR9, myeloid differentiation primary response gene 88 (MyD88), cytosolic RNA sensor MAVS or the purinergic receptor P2X7R maintain intact antitumor immunity responses, whereas mice deficient in STING or IRF3 present with impaired CD8^+^ T cell priming and activation [81, 82]. In fact, dying tumor cells can release multiple damage-associated molecular patterns (DAMPs) to trigger innate immune responses in DCs; among these released stimuli, tumor cell-derived DNA is a pivotal inducer. In general, the phagocytosis of apoptotic cells causes immune silence because of DNase-based degradation [66]. Nevertheless, tumor cell-released DNA can be preserved in the DC endolysosomal compartment through an unknown mechanism [66, 82]. cGAS recognizes DNA invading the cytoplasm and induces the activation of STING cascades, excretion of type I IFNs and expression of ISGs. Additionally, under some physiological conditions, such as hypoxia and acidic environments, nuclear or mitochondrial DNA might be packaged in exosomes. Exosomal DNA (ExoDNA) animates STING signaling once it is absorbed by tumor-infiltrating DCs [83]. Finally, tumor cell-derived cGAMP can also be transferred to host DCs by the folate transporter SLC19A1 and then directly binds to STING, activating it in DCs [84]. A recent study more directly demonstrated that cell-autonomous STING promoted the maintenance of stem cell-like CD8^+^ T cells and augmented antitumor T cell responses, and mechanistically, cGAS-STING-mediated type I interferon signaling reinforced the stem cell–like CD8^+^ T cell differentiation program mainly by restraining Akt activity [85].

**Fig. 2:**
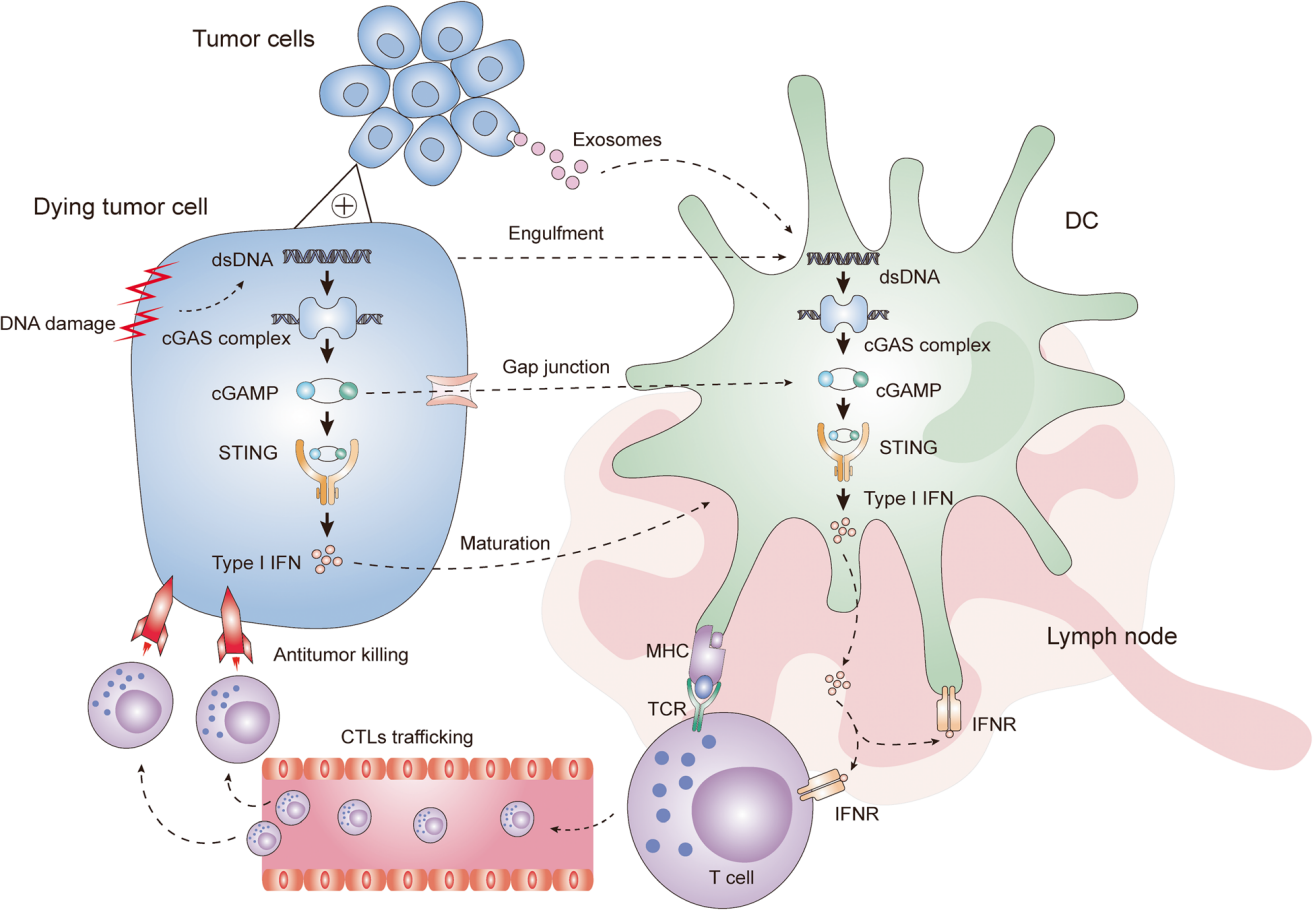
The antitumor immunity effect of the cGAS-STING pathway. DNA damage leads to the formation of dsDNA in tumor cells, upon its stimulation, STING signaling is activated and promotes the release of Type I IFN, which is crucial for DC maturation. STING signaling activation in DCs is the core step of the whole cancer-immunity cycle, which can be initiated through engulfment of dying/damaged tumor cells, exosome transfer and cGAMP gap junctions. Then DCs migrate towards the tumor-draining lymph node and cross-prime tumor specific CD8^+^ T cells with the help of Type I IFNs. Finally, T cells undergo clonal expansion and traffic through the blood vessel to conduct tumor killing

Immune cell-derived type I IFNs have crucial functions in antitumor immunity control. On the one hand, type I IFNs boost cross presentation by various mechanisms: first, they stimulate the maturation of DCs; second, they slow the endosome-lysosome acidification process to prevent engulfed tumor antigen clearance and elevate the expression of MHC I molecules on the cell surface [80, 86, 87]; finally, they accelerate DC migration towards lymph nodes, where they can cross-prime tumor-specific CD8^+^ T cells [88]. On the other hand, type I IFNs drive the expression of multiple chemokines, such as CXCL9 and CXCL10, both of which are necessary for cytotoxic T lymphocyte (CTL) transfer and infiltration [89]. Similarly, type I IFNs restrain the default immune suppressive action of regulatory T (T_reg_) cells by downregulating phosphodiesterase 4 (PDE4) and upregulating cyclic AMP (cAMP) [90]. Consequently, type I IFNs serve as bridges linking the cGAS-STING pathway with CD8^+^ T cell-mediated antitumor immunity. The antitumor mechanisms of the cGAS-STING signaling axis are illustrated in Fig. 2.

Indeed, previous studies revealed that STING activation can stimulate antitumor immune responses in leukemia, melanoma, glioma and hepatocellular carcinoma [91–94]. Additionally, STING expression is downregulated in a wide variety of tumor tissues and cell lines, according to a pan-cancer analysis, with a small proportion of tumors (approximately 1-25%) bearing silent STING expression [95]. Lower STING expression was found in hepatic carcinoma and gastric cancer compared with its level in corresponding normal tissues, and this lower expression level was correlated with higher tumor stage and poorer prognosis [96, 97]. Consistently, compared with that in the MCFG-10A mammary epithelial cell line, lower STING expression was detected in malignant breast cancer cell lines, including MCF-7, HBL100 and T47-D cells, as well as human melanoma cell lines and colorectal adenocarcinoma lines [96, 98, 99]. Collectively, these findings suggest that cGAS-STING signaling might act as a tumor suppressor in certain types of cancers.

## STING pathway agonists as cancer therapeutics

The immunostimulatory potential of the cGAS-STING pathway makes it an attractive pharmacological target, since its activation in the tumor microenvironment (TME) can induce efficient cross-priming of tumor-specific antigens and facilitate the infiltration of effector T cells. Recent drug research has focused on the development of STING agonists because of their potential in anticancer therapy [100, 101]. To date, various kinds of STING agonists have been discovered, and they are mainly divided into the following categories: cyclic dinucleotides and their derivates, DMXAA and its analogs, and small molecular agonists. In addition, some conventional antitumor therapeutics can also indirectly activate STING, such as chemotherapy, radiotherapy (RT), and targeted therapy [102]. In addition, STING agonists are able to enhance the efficacy of other anticancer therapeutic agents when used in combination. STING agonists and their synergistic use with other remedies is further explored in detail below.

### Cyclic dinucleotides (CDNs)

CDNs constitute a main type of STING agonist, which mainly originate from bacteria. The known natural CDNs consist of exogenous cyclic di-GMP (c-di-GMP), c-di-AMP, 3′3′-cGAMP and endogenous 2′3′-cGAMP. Among these, c-di-GMP, c-di-AMP, and 3′3′-cGAMP are synthesized by bacteria and identified as secondary messengers that mediate STING signal transduction in prokaryotic cells, while 2′3′-cGAMP functions as the initiator of STING in mammalian cells [103]. The antitumor potential of these natural dinucleotides was first proven by the finding that c-di-GMP could inhibit the proliferation of human colon cancer cells in vitro, and basal cell proliferation of human cecal adenocarcinoma (H508 cells) was inhibited with 50 μM c-di-GMP [104]. Intraperitoneal (i.p.) injection of high-dose c-di-GMP directly activated caspase-3 and triggered 4 T1 tumor cell apoptosis in vitro; 15 nmol of c-di-GMP reduced the growth of 4 T1 tumor cells in vitro by 70% and 150 nm reduced it by 92%, while low-dose c-di-GMP (0.01-2 nmol) accelerated the adaptive T cell response by converting a subgroup of myeloid-derived suppressor cells (MDSCs) into immune stimulatory cells producing IL-12 [105]. Consistently, i.p. injection of 3′3′-cGAMP (10 mg/kg) expedited dramatic leukemic elimination in El-TCL1 transgenic mice bearing chronic lymphocytic leukemia (CLL) and promoted tumor shrinkage of multiple myeloma in vivo [106]. From the perspective of endogenous CDNs, 2′3′-cGAMP (> 5 mg/kg) was also shown to restrain tumorigenesis and improve the survival rate of mice bearing CT26 colon adenocarcinoma in a dosage-dependent manner, relying on DC activation and T cell cross-priming [107]. More recently, Ohkuri, T. et al. further demonstrated that intratumoral (i.t.) injection of 2′3′-cGAMP (2.5 μg/25 μL/dose) on 5 and 10 days after the injection of tumor cells significantly mitigated tumor growth and prolonged the survival of breast cancer (4 T1-luc), squamous cell carcinoma (mSCC1), colon cancer (CT26), and melanoma (B16F10) mouse models [108]. Notably, the i.t. injection of 2′3′-cGAMP inhibited not only tumor growth but also lung metastases in mice bearing B16F10 cell-derived tumors, suggesting that cGAMP-induced CD8^+^ T-cell priming can drive systemic antitumor immunity to control local and distant tumor growth [109].

Considering the superior properties of STING signaling in activating adaptive immunity, it is rational to utilize STING agonists such as CDNs as cancer vaccine adjuvants to increase tumor immunogenicity [110]. Fu et al. investigated the in vivo therapeutic efficacy of a cancer vaccine termed STINGVAX, comprising granulocyte-macrophage colony-stimulating factor (GM-CSF) and bacteria-derived or synthetic CDNs. They observed that after i.t. injection of STINGVAX (with 20-200 μg of CDNs per vaccine dose), the volume of B16 melanoma tumors was dramatically reduced in a dose-dependent manner. Compared to mice receiving GM-CSF cancer vaccine alone, STINGVAX-treated mice had more infiltrating CD8^+^ IFN-γ^+^ T cells in the tumor microenvironment. The in vivo antitumor effect of STINGVAX was also verified in models of colon carcinoma (CT26), pancreatic carcinoma (Panc02) and upper aerodigestive squamous cell carcinoma (SCCFVII) [111].

Although natural CDNs are able to produce robust antitumor immunity, their chemical features might hinder their future application in the clinical setting. First, native CDNs are easily degraded by enzymes inside the cell or in the bloodstream. Second, their negatively charged property, hydrophilicity and phosphate moieties severely impede CDNs from penetrating cell membranes to activate cytosolic STING, leading to low bioavailability and poor retention of the CDNs in specific cells and tissues. Third, unintentional toxicities and narrow therapeutic windows are also unavoidable. Thus, new strategies to improve therapeutic efficacy and reduce adverse effects are urgently needed, including drug delivery carrier engineering, original structural modification and non-nucleotide agonist screening [112]. Regarding agonist delivery, Smith et al. reported that biopolymer implants codelivering c-di-GMP (6 μg) and chimeric antigen receptor T (CAR-T) cells resulted in significant tumor regression in mice bearing pancreatic tumors [113]. Moreover, intravenous (i.v.) administration of c-di-GMP/YSK05-Lip (equivalent to 3 μg of c-di-GMP), a YSK05-liposome delivery system encapsulating c-di-GMP, led to a tremendous decrease in metastatic lesions in a B16F10 mouse melanoma model, with nearly 40% of the injected mice showing resistance against tumor relapse, indicating that the adaptive immune response memory was successfully induced [114]. Chen et al. also found that intravenous (i.v.) injection of liposomal nanoparticle-delivered cGAMP (cGAMP-NP) could activate the STING axis more effectively than soluble cGAMP and converted the immunosuppressive TME to a tumoricidal state in a transplanted B16F10 cell melanoma model and in a genetically engineered triple-negative breast cancer model [115]. Moreover, a recent study creatively suggested that modified bacteria might be exploited as a selective carrier of STING agonists. Introduction of a di-nucleotide cyclase-coding gene into the *Escherichia coli* Nissle strain was an attempt at realizing this effect; however, advancements to the system are needed [102].

Apart from improving delivery methods, CDNs with superior properties are currently being synthesized and tested. For instance, to prevent enzymatic hydrolysis of cGAMP, the nonbridging oxygen atoms in cGAMP phosphodiester linkages were replaced by sulfur atoms. The modified compound, 2′3′-cGsAsMP, showed resistance against degradation by ENPP1, a major 2′3′-cGAMP hydrolase, thereby leading to a longer half-life and sustained high affinity for human STING (hSTING) [116]. Synthetic dithio mixed-linkage CDNs with both Rp, Rp (R, R) and Rp, Sp (R, S) dithio diastereomers possessed not only resistance to digestion by snake venom phosphodiesterase but also enhanced affinity for STING. A novel, superior modified product, ML RR-S2 CDA (also termed ADU-S100), had the potency to activate all hSTING variants and mouse STING (mSTING). ADU-S100 had higher efficiency in activating STING signaling than endogenous or exogenous CDNs, mainly because of its enhanced stability and lipophilicity. Its powerful tumor elimination effect was extensively demonstrated in multiple murine models, including B16 melanoma, 4 T-1 breast cancer and CT26 colon cancer, with all treated animals showing significant and durable tumor regression after i.t. injection of ADU-S100 (three 50 mg doses) when tumor volumes reached 100 mm^3^ [117]. The remarkable curative effect and high affinity for hSTING laid the foundation for its clinical use. Related clinical trials of ADU-S100 are outlined in Table 3. In addition to ADU-S100, some other novel STING agonists have been well designed. IACS-8779 and IACS-8803 are two highly potent 2′3′-thiophosphate CDN analogs that induced striking systemic antitumor responses in a B16 melanoma murine model after i.t. injection (10 μg on 6, 9 and 12 days posttumor implantation) compared with ADU-S100 or cGAMP [118]. The characteristics and preclinical applications of all these mentioned CNDs are summarized in Table 2. Because of the structural modification and optimization of delivery strategies, the application range and efficacy of CDNs have been dramatically expanded, and new agonists with better properties are expected to emerge.

**Table 2:** Characteristics and preclinical applications of different STING agonists

Classification		Characteristics	Application models	Treatment information	Therapeutic effects	References
Natural CDN agonists	c-di-GMP	Poor membrane permeability; suitable for various codelivery technologies	Colon cancer (H508 cells);	50 μM	Inhibits proliferation	[104, 105]
			4 T1 metastatic breast cancer	15 nmol (i.p.)	70% tumor regression	
				150 nmol (i.p.)	92% tumor regression	
				0.01-2 nmol (i.p.)	Accelerates T-cell response	
	3′3′-cGAMP	Higher binding affinity for mSTING than for hSTING	Chronic lymphocytic leukemia;	10 mg/kg (i.p.)	Leukemia elimination	[106]
			multiple myeloma	10 mg/kg (i.p.)	Suppresses growth	
	2′3′-cGAMP	Higher affinity for hSTING than its lineage isomers; binds to various STING nucleotide polymorphisms observed in humans; easily degraded by phosphodiesterase; impermeable to the cell membrane	CT26 colon adenocarcinoma;	> 5 mg/kg	Restrains tumorigenesis;	[107, 108]
					Improves survival rate	
			breast cancer (4 T1-luc);	2.5 μg/25 μL/dose (i.t.)	Delays tumor growth	
			squamous cell carcinomas (mSCC1);	2.5 μg/25 μL/dose (i.t.)	Delays tumor growth	
			colon cancer (CT26);	2.5 μg/25 μL/dose (i.t.)	Delays tumor growth	
			melanoma (B16F10)	2.5 μg/25 μL/dose (i.t.)	Delays tumor growth	
Synthetic CDN agonists	STINGVAX	Potent in vivo antitumor efficacy in multiple therapeutic models of established cancer	B16 melanoma;	20-200 μg CDNs (i.t.)	Reduces tumor volume	[104]
			colon carcinoma (CT26);			
			pancreatic carcinoma (Panc02)			
	cGAMP-NPs	Liposomal nanoparticles (NPs) deliver cGAMP intracellularly more effectively than realized with soluble cGAMP	B16F10 melanoma;	i.v.	Creates a tumoricidal state	[108]
			TNBC			
	Biopolymer scaffolds (c-di-GMP and CAR T cells)	Eradicates tumors more effectively than systemic delivery	Pancreatic cancer	6 μg c-di-GMP	Tumor regression	[106]
	c-di-GMP/YSK05-Lip	YSK05 is a lipid that can efficiently deliver c-di-GMP to the cytosol; possesses high fusogenic activity, which enhances endosomal escape	B16F10 mouse melanoma	3 μg c-di-GMP (i.v.)	Decreases metastasis	[107]
	2′3′-cGsAsMP	More resistant to degradation by ENPP1; ten-fold more potent at inducing IFN secretion; potential use as a cancer vaccine adjuvant	THP1 monocytes			[116]
	ADU-S100	Improves stability and lipophilicity; higher affinity for hSTING than natural CDN agonists; capable to activate all hSTING variants and mSTING	B16 melanoma;	three 50 mg doses (i.t.)	Durable tumor regression	[117]
			4 T-1 breast cancer;	three 50 mg doses (i.t.)	Durable tumor regression	
			MC26 colon cancer	three 50 mg doses (i.t.)	Durable tumor regression	
	IACS-8779	Stimulates a superior systemic antitumor response than ADU-S100 and cGAMP	B16 melanoma	10 μg on day 6, 9 and 12 posttumor implantation	Antitumor response	[118]
	IACS-8803					
Non-CDN agonists	FAA	Causes hemorrhagic necrosis; failed in a phase I clinical trial due to species specificity	Murine colon tumors		Extensive tumor rejection	[119, 120]
	DMXAA	First discovered as a vascular disrupting agent; high affinity for mSTING, with minimal effect on hSTING	Rat mammary carcinoma;	300 mg/kg (i.p.)	High anticancer potency	[91, 117, 121–123]
			B16 melanoma;	500 μg (i.t.)	Accelerates tumor rejection	
			NETs;	22.5 mg/kg (i.p.)	Inhibits tumor growth	
			acute myeloid leukemia;	450 μg (i.v.)	Inhibits tumor growth	
			glioma GL261;	25 mg/kg (i.p.)	Inhibits tumor growth	
			lung cancer	25 mg/kg (i.p.)	Inhibits tumor growth	
			adrenocortical cancer	22.5 mg/kg (i.p.)	Inhibits tumor growth	
	α-Mangostin	Higher affinity for hSTING than for mSTING	THP1 cells			[124]
	CMA	Exclusive mSTING agonist; inactive against human cells	HEK 293 T cells; mouse macrophages		Antiviral activity	[125]
Indirect agonists	Radiotherapy	Causes the release of cytosolic DNA fragments; low RT doses decrease TREX1, thus activating STING	MC38 colon cancer;	20 Gy	Adaptive immune response;	[126]
			HT29 colorectal tumor cells	6 Gy	induces type III IFNs	[127]
	Cisplatin	Forms DNA adducts and inhibits DNA repair	Epithelial ovarian cancer	11.8 μM	Accumulates T cells	[128]
	Teniposide	Activates STING; increases MHC expression on the tumor cell surface	B16 melanoma;		Activates DC and T cells	[129]
			colon cancer (CT26, MC38)			
	PARPi (Olaparib)	Generates cytoplasmic chromatin fragments with characteristics of micronuclei	ERCC1-deficient NSCLC cells;		Inhibits proliferation	[130]
			Brca1-deficient ovarian cancer;	50 mg/kg/day (i.p.)	Strong T-cell cytotoxicity	[126]
			Brca1-deficient TNBC	50 mg/kg/day (i.p.)	Strong T-cell cytotoxicity	[131]
	CHK1i (Prexasertib)	Accelerates DNA double-strand breaks and STING activation	SCLC tumors	10 mg/kg twice daily	Enhances T-cell recruitment	[132]
Small-molecule agonists	C11	Triggers IRF3/IFN-dependent responses in a STING-dependent manner	THF cells		Blocks replication of alphavirus	[133]
	BNBC	Induces innate immunity against various viruses and promotes the activation of adaptive immune responses	Primary human fibroblasts and PBMCs		Antiviral activity	[134]
	DSDP	Induces proinflammatory cytokines in a STING-dependent manner	Human fibroblasts		Antiviral activity	[135]
	G10	Selectively induces STING-dependent synthesis and secretion of bioactive IFNs; no evidence of binding directly to STING	Human fibroblasts		Antiviral activity	[136]
	ABZI	Activates STING in “open” conformation; sub-micromolar levels induce STING activation and IFN production	Colon tumors	1.5 mg/kg (i.v.)	80% of a treated group remained tumor free	[52]

*FAA,* flavone acetic acid; *DMXAA,* 5,6-dimethylxanthenone-4-acetic acid; *CMA,* 10-carboxymethyl-9-acridanone; *PARPi,* PARP inhibitor; *CHK1i,* CHK1 inhibitor; *C11,* N-(Methylcarbamoyl)-2-{[5-(4-methylphenyl)-1,3,4-oxadiazol-2-yl]sulfanyl}-2-phenylacetamide; *BNBC,* 6-bromo-N-(naphthalen-1-yl)-benzo [d ][1, 3] dioxole-5-carboxamide; *DSDP,* dispiro diketopiperazine; *G10,* 4-(2-chloro-6-fluorobenzyl)-N-(furan-2-ylmethyl)-3-oxo-3,4-dihydro-2H-benzo [b]thiazine-6-carboxamide; *ABZI,* amidobenzimidazole; *TNBC:* Triple-Negative Breast Cancer; *PBMC:* Peripheral blood mononuclear cell;* i.t.: *intratumoral injection; *i.p.:* intraperitoneal injection; *i.v.:* intravenous injection

### DMXAA and its analogs

Non-CDN STING agonists are also being researched. Flavone acetic acid (FAA), an antitumor agent, has recently been identified as a mSTING agonist, which may result in the extensive rejection of murine colon tumors through hemorrhagic necrosis [137]. However, it failed in a phase I clinical trial and showed no effect in rat tumor models [119], probably due to its species specificity. In an attempt to obtain structurally similar compounds that can also induce tumor hemorrhagic necrosis, the structure of FAA was modified, and several analogs were generated. The flavone-8-acetic acid derivative 5,6-dimethylxanthenone-4-acetic acid (DMXAA) has been the most systematically studied [138]. Intraperitoneal injection of DMXAA (300 mg/kg) elicited high anticancer potency against rat mammary carcinoma [139] and showed antitumor functions in multiple mouse models (Table 2). Seven days after tumors were inoculated, i.t. injection of 500 μg DMXAA accelerated the tumor abrogation in STING-expressing mice bearing B16 melanoma tumors, while STING-null mice were less responsive [117]. The inhibitory effects of DMXAA on cancer growth were also confirmed in mouse models of gastroenteropancreatic neuroendocrine tumors (NETs) (i.p., 22.5 mg/kg), adrenocortical cancer (i.p., 22.5 mg/kg) [121], acute myeloid leukemia (i.v., 450 μg) [91], glioma (GL261) (i.p., 25 mg/kg) [122] and lung cancer (344SQ-ELuc) (i.p., 25 mg/kg) [123]. Unfortunately, DMXAA failed in clinical trials since its interaction was restricted to mSTING [140]. Another similar derivative named 10-carboxymethyl-9-acridanone (CMA) was also identified as an exclusive mSTING agonist [125]. Despite the clinical failures, these valuable works spurred efforts to design novel non-nucleotide analogs with higher affinity for hSTING. To this end, Gao et al. designed C7-functionalized DMXAA derivatives but without significant success [141]. However, Quan et al. revealed that the DMXAA derivative α-mangostin was more efficient in activating hSTING than in activating mSTING [124]. These findings suggest that the rational design of DMXAA analogs will inspire the emergence of novel antitumor therapies.

### Indirect STING agonists

The antitumor immunity function of direct STING agonists has been widely explored, but most STING agonists have limited clinical application due to their poor druggability. Thus, developing new STING agonists that are effective, safe and easy to administer remains a challenge. Some classical anticancer treatments originally designed for other intended mechanisms, such as radiotherapy, chemotherapy, and targeted therapy, might activate the STING pathway (Table 2) [102]. Indeed, irradiated tumor cells released genome fragments into the cytoplasm directly or shuttled self-DNA encapsulated in exosomes to host immune cells to prime STING activation and adaptive immune responses [83, 142]. High doses of radiation (20–30 Gy) elevated the expression of three prime repair exonuclease 1 (TREX1), a DNA nuclease that mainly degrades cytoplasmic DNA, to clear cytoplasmic dsDNA, while low doses of radiation therapy prevented TREX1 activation, thus stimulating DNA fragment release to activate the STING pathway [143]. For example, radiotherapy (20 Gy) evoked innate immune sensing dominated by the STING pathway in a MC38 tumor murine model, further driving the adaptive immune response to radiation [126]. Exposure to gamma rays (6 Gy) directly induced type III IFNs mediated by the STING signaling axis in HT29 colorectal tumor cells [127]. Chemotherapies can induce DNA damage and inhibit DNA repair simultaneously; damaged DNA then activates the cGAS-STING axis to potentiate DC-mediated antigen presentation and T-cell priming [129, 144]. Chronic cisplatin treatment (11.8 μM) promoted intratumoral T-cell accumulation and advanced tumor immunogenicity through the cGAS-STING pathway in an epithelial ovarian cancer mouse model [128]. Teniposide treatment facilitated MHC expression on the tumor cell surface and activated DCs and T cells in a STING-dependent manner [129]. Additionally, targeted therapies also possess the capacity to augment STING-mediated immune responses. For example, PARP inhibitor (PARPi) olaparib treatment generated cytoplasmic chromatin fragments with characteristics of micronuclei to promote cGAS-STING activation and downstream CCL5 secretion in ERCC1-deficient non-small cell lung cancer cells [130]. In addition, olaparib (i.p., 50 mg/kg daily) also elicited strong T cell-mediated cytotoxicity in a Brca1-deficient ovarian cancer mouse model and a BRCA1- and TP53-deficient genetically engineered mouse model (GEMM) of triple-negative breast cancer (TNBC) [131, 145]. The efficacy of olaparib was abolished when TBK1 inhibitors were coadministered to mouse ovarian tumors, which verified that intact STING signaling was indispensable for PARPi efficacy [126]. Moreover, the CHK1 inhibitor (CHK1i) prexasertib (10 mg/kg twice daily) also accelerated DNA double-strand breaks and STING activation, subsequently enhancing T-cell recruitment and effector cell function in small cell lung cancer (SCLC) mouse tumors [132]. Therefore, conventional cancer therapies can mediate immune responses by modulating STING activation, which may expand their clinical application and promote their future use in combination with other drugs.

Some novel small-molecule compounds were also found to be indirect STING agonists (Table 2). Bryan Gall et al. conducted a high-throughput screening assay and identified a compound named N-(Methylcarbamoyl)-2-{[5-(4-methylphenyl)-1,3,4-oxadiazol-2-yl]sulfanyl}-2-phenylacetamide (referred to as C11), which triggered IFN-mediated antiviral immune responses in a STING-dependent manner in THF cells [133]. Moreover, 6-bromo-N-(naphthalen-1-yl)-benzo [d ][1, 3] dioxole-5-carboxamide (also referred to as BNBC) was reported to initiate innate immunity against a broad spectrum of viruses and adaptive immune responses in primary human fibroblasts and peripheral-blood mononuclear cells (PBMCs) [134]. Another compound, dispiro diketopiperazine (DSDP) and 4-(2-chloro-6-fluorobenzyl)-N-(furan-2-ylmethyl)-3-oxo-3,4-dihydro-2Hbenzo[b]thiazine-6-carboxamide (referred to as G10), facilitated the selective secretion of proinflammatory cytokines from human fibroblasts in a STING-dependent manner [136]. No sufficient evidence indicates that the aforementioned small-molecule agonists bind directly to STING [135]. A recent study revealed that i.v. injection of amidobenzimidazole (ABZI) (1.5 mg/kg) in immunocompetent mice with established syngeneic colon tumors was able to induce tumor regression, with nearly 80% of the treated group remaining tumor free at the end of the study, and this strong inhibition effect was reversed by CD8^+^ T-cell depletion [52].

### Combinations with other therapeutics

STING agonists are considered ideal sensitizers for immune checkpoint inhibitors. First, preexisting CTLs are prerequisites for effective immune checkpoint inhibitor (ICI) treatment. Most tumors are insensitive to ICIs due to a lack of T-cell infiltration, but these “cold tumors” can be transformed into “hot tumors” by STING agonist-mediated T-cell priming and infiltration. This process might be related to the expression of IFN-stimulated genes such as CXCL9 and CXCL10 [146]. Therefore, interventions supporting T-cell infiltration are conducive to alleviate ICI resistance. Second, the STING signaling axis enhances the susceptibility of tumor cells to immune attack by natural killer (NK) cells and CTLs [147]. Third, STING pathway activation is accompanied by the upregulation of several immune inhibitory factors, including PD-L1, IDO, and FOXP3, resulting in immune suppression and failed spontaneous tumor elimination [148]. The combination of STING agonists with ICIs such as CTLA-4 and PD-1 blockers not only can neutralize the immunosuppressive effect of STING agonists but can also sensitize cells to ICIs. Indeed, the synergistic combination of RR-CDG (i.t., every 3 days) with a PD-L1 blockade (i.p.,100 μg) stimulated stronger antitumor responses than monotherapy in a head and neck squamous cell cancer (HNSCC) mouse model [149]. Intramuscular (i.m.) delivery of cGAMP (1-10 μg) into a B16 melanoma mouse model strongly enhanced the antitumor effect of the PD-L1 blockade (i.p., 200 μg) [150]. Furthermore, the coadministration of STINGVAX (subcutaneous injection of 20 μg of CDN per vaccine dose) with a PD-1 blocker (i.p., 200 μg, twice a week) prompted the regression of poorly immunogenic tumors that were not responsive to anti-PD1 monotherapy [111]. Complete tumor regression and long-term antitumor memory were formed in mice bearing TC-1 tumors after they received a combination therapy consisting of a STING-activating nanovaccine and PD-1 blockade [151]. More surprisingly, a therapeutic regimen containing the STING agonist MK-1454 (i.t. injection weekly for 9 weeks and then every 3 weeks) and pembrolizumab (i.v., 200 mg every 3 weeks) have entered clinical tests for solid tumors and lymphomas (Table 3). As previously mentioned, some conventional antitumor therapies can stimulate STING-mediated adaptive immunity and may sensitize these tumors to other drugs. Treatment with olaparib (i.p., 50 mg/kg/day) and anti-PD1 antibody (i.p., 250 μg/mouse every 3 days) led to sustained tumor growth control and extended survival time for BRCA-deficient ovarian cancer models [145]. The combination of PARPi (BMN673) (oral gavage, 0.33 mg/kg/day) and anti-PDL1 antibody (i.p., 200 μg/mouse, every 3 days) significantly mitigated the tumor burden in colorectal and ovarian syngeneic and nude mouse models [152]. In addition to synergizing anti-PD1 therapy, intact STING signaling is also indispensable for the antitumor effects of the CTLA-4 checkpoint blockade, as evidenced by the finding that mice grafted with STING-deficient B16 tumors showed almost no tumor elimination after receiving a combination treatment of irradiation and anti-CTLA-4 antibody. More obviously, in a prostate cancer murine model, a combination cocktail containing anti-CTLA-4 antibody (i.p., 100 μg/mouse), anti-PD-1 antibody (i.p., 250 μg/mouse), anti-4-1BB antibody (i.p., 200 μg/mouse) with the STING agonist CDG (i.t., 25 μg/mouse) caused unprecedented tumor regression in 75% of the mice, demonstrating a higher cure rate than ICI monotherapy [153]. CAR-T cell therapy was used in combination with cdGMP in a pancreatic tumor mouse model, with the results showing complete tumor elimination in approximately 40% of the treated mice [113].

**Table 3:** Feasible combination therapies in human clinical trials

Treatment regimens	Cancer type	Phase	Status	Locations	NCT
ADU-S100 + Anti-PD antibody	Metastatic/Recurrent Head and Neck Cancer	II	Recruiting	United States	NCT03937141
ADU-S100 + Anti-PD antibody	Advanced/Metastatic Solid Tumors or Lymphomas	I	Active, not recruiting	United States	NCT02675439
MK-1454 + Pembrolizumab	Solid Tumors and Lymphoma	I	Recruiting	United States	NCT03010176
DMXAA + Taxane-based chemotherapies	Solid Tumor Malignancies	I	Terminated	United States	NCT01290380
DMXAA + Docetaxel	Advanced or Recurrent Solid Tumors	I	Completed	Japan	NCT01285453
DMXAA + Paclitaxel and Carboplatin	Non-Small Cell Lung Cancer	III	Terminated	United States	NCT00662597
DMXAA + Cetuximab	Refractory Solid Tumors	I	Withdrawn	United States	NCT01031212
DMXAA + Docetaxel	Urothelial Carcinoma	II	Withdrawn	United States	NCT01071928
DMXAA + Paclitaxel and Carboplatin	Non-Small Cell Lung Cancer	I	Completed	Japan	NCT00674102

Growing evidence also recommends STING agonists as adjuvants with common antitumor therapies such as chemotherapy, radiotherapy and targeted therapy. Combination therapy using cisplatin (i.p., 6 mg/kg) and cGAMP (i.t., 2.5 μg) showed potent antitumor effects in a CXCR3-dependent manner in mouse squamous cell carcinoma models [154]. The combined administration of 5-fluorouracil (5-FU) (i.p., 10 mg/kg/day) with cGAMP (i.t., 5 mg/kg/day) in a CT26 colon cancer murine model ameliorated tumor progression and reduced the intestinal side effects of the 5-FU treatment [107]. Similarly, i.t. injection of 2′3′-cGAMP (10 μg) significantly synergized the antitumor effect of radiation (20 Gy) in a STING-dependent manner in the MC38 tumor models, compared with the effect of monotherapy [126]. Congruent results were also observed in Panc02 pancreatic adenocarcinoma when radiotherapy (10 Gy) was combined with the STING agonist RR-S2-CDG (10 μg); this combination regimen generated the T-cell immunity required for the control of local tumors and distant metastasis [155]. In terms of targeted therapies, combined treatment with cetuximab and STING agonists facilitated tumor recession in patients with HPV-positive (HPV^+^) head neck squamous cell carcinoma (HNSCC), which may depend on cetuximab-mediated NK cell activation and DC maturation [156]. Additionally, Pei et al. demonstrated that the STAT3 inhibitor HJC0152 (i.t., 30 μg) also enhanced the treatment effect of the STING agonist c-di-AM (PS)2 (i.t., 10 μg), leading to significant tumor rejection in a 4 T1 breast cancer model [157]. Details of all the aforementioned combination treatment regimens are available in Table 4.

**Table 4:** Feasible combination therapies applied to preclinical tumor models

Treatment regimens		Cancer type	Therapeutic effects	References
Immunotherapy-STING agonists combination	RR-CDG (i.t., every 3 d) + PD-L1 blockade (i.p., 100 μg)	HNSCC	Stronger antitumor effects than monotherapy	[149]
	cGAMP (i.m., 1-10 μg) + PD-L1 blockade (i.p., 200 μg)	B16 melanoma	Augments antitumor effect	[150]
	STINGVAX (subcutaneous injection 20 μg CDN per vaccine dose) + PD-1 blockade (i.p., 200 μg twice a week)	CT26 colon cancer	Regression of poorly immunogenic tumors	[111]
	STING-activating nanovaccine + PD-1 blockade	TC-1 tumor models	Complete tumor regression	[151]
	PBAE-CDN (i.t., 2 μg) + anti-PD-1 antibody (i.p., 100 μg twice weekly)	B16 melanoma tumors	Significantly reduces tumor growth compared to unencapsulated CDNs	[158]
	Olaparib (i.p., 50 mg/kg/d) + anti-PD-L1 antibody (i.p., 250 μg every 3 days)	BRCA-deficient ovarian cancer	Sustained tumor growth control and extended survival	[126]
	BMN673 (oral gavage, 0.33 mg/kg/d) + anti-PD-L1 antibody (i.p., 200 μg, every 3 days)	Colorectal and ovarian cancer	Reduces tumor burden	[152]
	Teniposide (i.p., 10 mg/kg) + anti-PD1 antibody (i.p., 100 μg, every 3 days)	B16 melanoma; colon cancer	Potentiates efficacy of anti-PD1 therapy	[129]
	Irradiation + anti-CTLA-4 blockade	B16 melanoma	Significant reduction in the growth of abscopal tumors	[70]
	ICI cocktail (i.p., 100 μg) + CDG (i.t., 25 μg)	Prostate cancer	Higher cure rate than monotherapy	[153]
	cdGMP + CAR-T cells	Pancreatic tumors	Approximately one-half of the treated mice had complete tumor elimination	[113]
Chemotherapy-STING agonists combination	cGAMP (i.t., 2.5 μg) + cisplatin (i.p., 6 mg/kg)	Squamous cell carcinoma	Antitumor effects	[154]
	cGAMP (i.t., 5 mg/kg/d) + 5-FU (i.p., 10 mg/kg/d)	CT26 cancer	Ameliorates tumor progression	[107]
Radiotherapy-STING agonists combination	2′3′-cGAMP (10 μg) + radiotherapy (20 Gy)	MC38 tumors	Significant antitumor effect	[126]
	RR-S2-CDG (10 μg) + radiotherapy (10 Gy)	Panc02 pancreatic adenocarcinoma	Controls local tumors and distant metastasis	[155]
Targeted therapy-STING agonists combination	cGAMP + cetuximab	HPV^+^ HNSCC	Tumor recession	[156]
	c-diAM (PS)2 (i.t., 10 μg) + STAT3 inhibitor (i.t., 30 μg)	Mouse 4 T1 tumors	Significant tumor regression	[157]
	STING agonist (50 μg) + GITR antibody (100 μg)	B-cell lymphoma	Synergistic antitumor effects	[159]

*HNSCC,* head and neck squamous cell cancers; *STINGVAX,* granulocyte-macrophage colony-stimulating factor (GM-CSF) with CDNs; *PBAE-CDN,* poly (beta-amino ester) cyclic dinucleotide; *DMXAA,* 5,6-dimethylxanthenone-4-acetic acid;* i.t.:* intratumoral injection; *i.p.:* intraperitoneal injection; *i.m.:* intramuscle injection

Nonetheless, not all tumors are responsive to STING agonists, especially those with tolerogenic DNA and low tumor antigenicity [160]. For tumors lacking sufficient cGAS or STING, such as melanoma, colorectal and alternative lengthening of telomeres (ALT)-related cancer, targeting the cGAS-STING pathway for antitumor therapy may not be feasible, and oncolytic virus treatment might represent an alternative approach [161].

## The protumor role of the cGAS-STING pathway

**Fig. 3:**
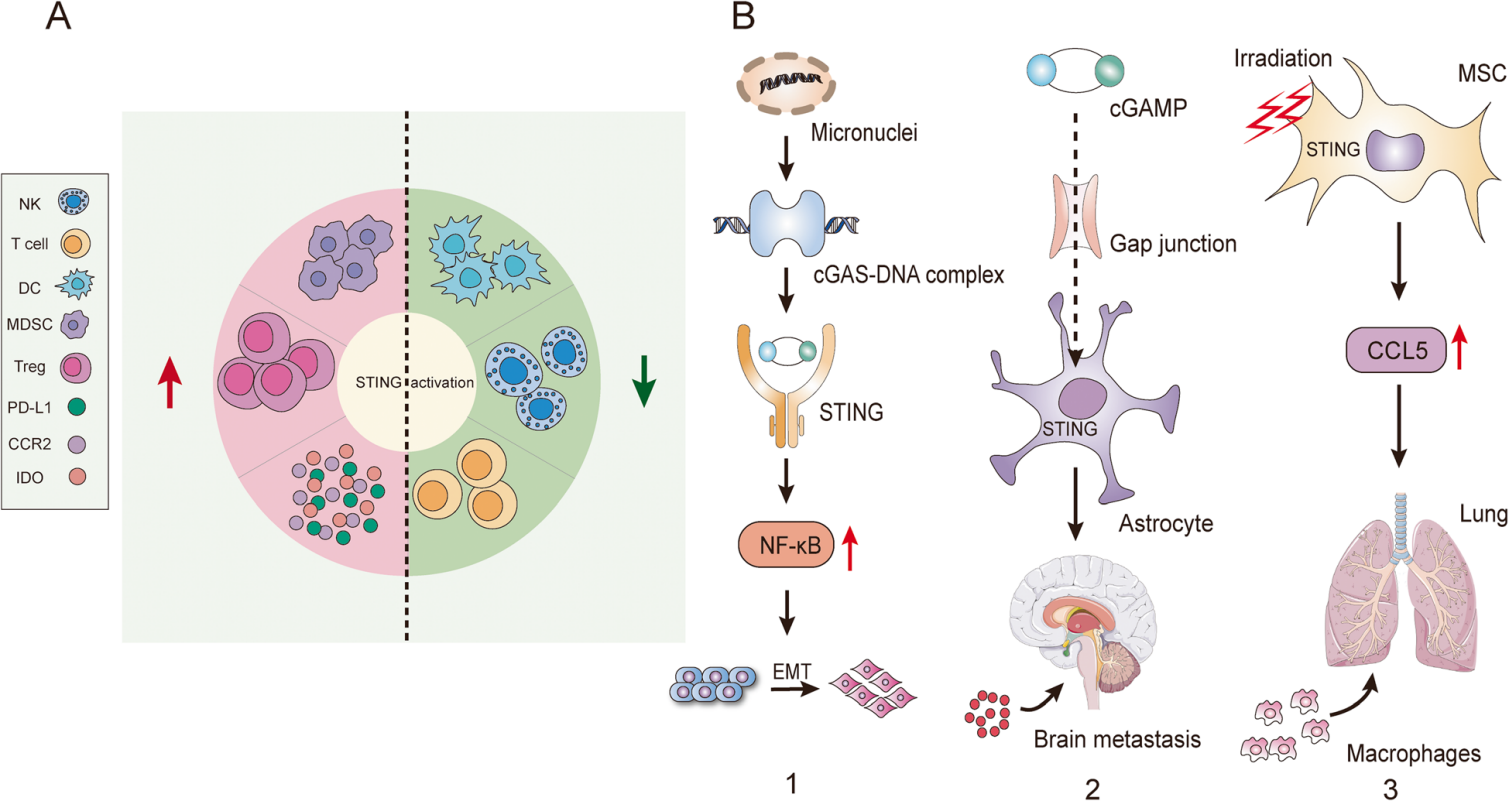
The protumor role of the cGAS-STING signaling pathway. **A** Chronic activation of STING creates an immunosuppressive tumor microenvironment. Continuous STING activation in turn impedes the original antitumor immunity by increasing the infiltration of MDSCs, Tregs and expression of PD-L1, IDO and CCR2, while decreasing the infiltration of NK cells, DCs and T cells. **B** The function of the STING signaling in tumor metastasis. **B-1** delineates the cell autonomous pro-metastasis effect, and **B-2**, **B-3** depict the cell nonautonomous pro-metastasis effect

Emerging evidence reveals that the cGAS-STING signaling cascade may have dichotomous effects on tumor development [66]. Highly aggressive and unstable tumors can paradoxically coopt cGAS-STING signaling to stimulate carcinogenesis. Accordingly, STING agonists should be carefully used with the contextual consideration of specific tumor stage, genotype and CIN [61]. In general, STING signaling is involved in malignant transformation mainly by creating an immune suppressive tumor microenvironment and promoting tumor metastasis (Fig. 3).

### STING participates in immunosuppression

With chronic activation of STING signaling, a potential immune suppressive landscape is generated [61, 162]. For example, 7,12-dimethylbenz(a) anthracene (DMBA) leads to skin tumorigenesis by inducing nuclear DNA leakage and STING activation. Bone marrow transplant experiments further revealed that STING expression in hematopoietic stem cells is vital for DMBA-induced skin tumorigenesis [163]. STING activation in human HPV^+^ tongue squamous cell carcinoma (TSCC) could facilitate the infiltration of regulatory T cells (Tregs), and enriched Tregs in turn released IL-10 to restrain the activity of antigen-specific T cells, ultimately promoting tumor progression [164]. In another study, exposure of MC38 mouse colon tumors to radiation led to the mobilization of myeloid-derived suppressor cells (MDSCs) in a STING-dependent manner. An analysis of TCGA data sets also revealed that STING expression is positively correlated with the infiltration of almost all immune cells, including MDSCs and Tregs, in pan-cancer, especially in bladder urothelial carcinoma, breast cancer, liver hepatocellular carcinoma, prostate adenocarcinoma and thyroid carcinoma [165]. An immune suppressive TME is characterized by the upregulated expression of the immune checkpoint indoleamine 2,3-dioxygenase (IDO), a symbol of tumor immune evasion. However, in STING-knockout mice, significantly decreased IDO levels and MDSCs were detected in the TME of a Lewis lung carcinoma (LLC) mouse model. Further, LLC growth promoted by intact STING would be alleviated when IDO expression was suppressed [160]. Hence, immune inhibitory cells and IDO in the TME are required for the STING signaling -involved tumorigenesis [160, 166]. In these cases, STING-triggered IDO expression must be detected before STING agonists are applied, and combining IDO inhibitors with STING agonists may counteract detrimental effects. In addition, STING activation also enhanced the expression of PD-L1 and CCR2 in multiple mouse tumors, including colon cancer, tongue squamous carcinoma, and head and neck squamous cell carcinoma [111, 167]. High expression of CCR2 in MDSCs led to the aggregation of tumor-promoting monocytes, which was prevented by the simultaneous use of CCR2 antagonists [168]. Surprisingly, in contrast to the findings described above, STING activation also promoted the death of the T-cells expressing it, possibly due to unresolved ER stress [169]. The accumulation of T cells was observed in the spleen and lymph nodes of STING-deficient mice [170]. In another experiment, STING expression was abrogated in T cells in a mixed bone marrow chimera model, while a significant increase in Ki67+ CD8+ memory T cells was found under STING-deficient conditions compared with the level in the untreated group [169, 170]. A series of studies suggested that STING activation in T cells may directly impair the adaptive immune system and accelerate tumorigenesis. Additional studies will be required to further delineate the explicit mechanism by which host STING activity facilitates a potent immune suppressive environment.

### An emerging characteristic of the STING pathway: prometastasis

STING signaling is closely related to tumor metastasis in both a cell autonomous and nonautonomous manner [171]. On the one hand, chromosomal instability induced micronuclei formation and STING activation to trigger noncanonical NF-κB signaling and inflammatory responses, which favored the epithelial-to-mesenchymal transition and metastasis [171]. On the other hand, tumor cell metastasis may be driven by nonautonomous mechanisms. Intriguingly, facilitated by the connexin 43 protein (CX43) and protocadherin 7 (PCDH7), the cGAMP produced in tumor cells was exported to adjacent astrocytes via gap junctions [172]. In this way, STING was activated in astrocytes and subsequently initiated the release of inflammatory cytokines and the activation of STAT1 and NF-κB, leading to brain metastasis of breast cancer and lung cancer [172]. Irradiated mesenchymal stromal cells (MSCs) also acquired the capacity to facilitate lung metastasis of breast cancer cells through cGAS-STING activation, and STING-mediated CCL5 expression increased the recruitment of macrophages for lung colonization [173].

Taken together, pieces of convincing evidence has revealed the dichotomous effects of the cGAS-STING pathway, although the complete mechanistic relevance of cGAS-STING signaling with respect to tumor development remains to be clarified. Therefore, we must extensively fine-tune the application range of STING agonists to avoid potential unintended adverse effects when used in the clinic.

## Concluding remarks

In addition to the canonical role of cGAS-STING signaling in antimicrobial innate immunity, emerging evidence has proven that its activation also stimulates antitumor immune responses. These meaningful findings have spurred efforts to harness this natural defense-related pathway in the next generation of cancer immunotherapy. STING agonists have a remarkable ability to promote CD8^+^ T cell infiltration, which indicates that they can be utilized in combination with other therapeutic modalities for the effective treatment of cancer or other diseases. In vivo studies revealed that STING agonists can enhance the efficacy of multiple therapies, including but not limited to chemoradiotherapy, targeted therapy and immunotherapy, confirming that the modulation of the cGAS-STING signaling pathway may serve as a promising anticancer treatment. Notably, as a key inducer of type I IFN responses, the STING pathway that is chronically activated can instigate tumor growth and metastasis, an effect that is related to tumor stage, CIN state, and degree of STING activation. Hence, therapeutic windows and the tumor status must be carefully evaluated before the application of STING agonists in clinical practice. Moreover, another dilemma should be deliberated at this time: activation of STING can recruit both immune-supporting cells to inhibit malignant transformation and immunosuppressive cells to drive tumor progression. Unfortunately, which immune modulation will dominate in the context of different tumor types remains unknown. Further endeavors are warranted to reveal the underlying mechanisms of STING-mediated immune responses in specific tumors. Nevertheless, in-depth knowledge of the cGAS-STING signaling axis indeed represents exciting progress in the field of cancer immunology and clinical treatment of cancer.

## Data Availability

Not applicable.

## Abbreviations

cGAS: Cyclic GMP-AMP synthase
STING: Stimulator of interferon genes
IFNs: Type I interferons
PRRs: Pattern-recognition receptors
PAMPs: Pathogen-associated molecular patterns
RIG-I: Retinoid acid inducible gene I
TLR9: Toll-like receptor 9
AIM2: AIM2-like receptor
2′3′-cGAMP: 2′3′ -cyclic GMP-AMP
ssDNA: Single-chain DNA
ER: Endoplasmic reticulum
STIM1: Stromal interaction molecule 1
LBD: Ligand binding domain
COPII: Coat protein complex II
ARF: ADP-ribosylation factor
TBK1: TANK-binding kinase 1
IRF3: Interferon regulatory factor 3
ISGs: Interferon-stimulated genes
IKK: IκB kinase
NF-κB: Nuclear factor-κB
HSV1: Herpes simplex virus 1
HSV2: Herpes simplex virus 2
KSHV: Kaposi sarcoma–associated herpesvirus
HIV: Human immunodeficiency virus
SIV: Simian immunodeficiency virus
VSV: Vesicular stomatitis virus
CDNs: Cyclic dinucleotides
SARS-COV-2: Severe acute respiratory syndrome coronavirus 2
CIN: Chromosomal instability
ExoDNA: Exosomal DNA
SASP: Senescence-associated secretory phenotype
BAX: BCL2-associated X
CAC: Colitis-associated carcinomas
AOM: Azoxymethane
DSS: Dextran sulfate sodium
DC: Dendritic cell
BATF3: Basic leucine zipper transcription factor ATF-like 3
DAMP: Damage-associated molecular pattern
CTL: Cytotoxic T lymphocyte
T_reg_: Regulatory T
PDE4: Phosphodiesterase 4
cAMP: Cyclic AMP
TME: Tumor microenvironment
MDSC: Myeloid-derived suppressor cells
CLL: Chronic lymphocytic leukemia
GM-CSF: Granulocyte-macrophage colony-stimulating factor
CAR-T: Chimeric antigen receptor T
cGAMP-NP: Nanoparticle-delivered cGAMP
hSTING: Human STING
mSTING: Mouse STING
FAA: Flavone acetic acid
NETs: Gastroenteropancreatic neuroendocrine tumors
CMA: 10-carboxymethyl-9-acridanone
TREX1: Three prime repair exonuclease 1
PARPi: PARP inhibitor
GEMM: Genetically engineered mouse model
TNBC: Triple-negative breast cancer
SCLC: Small cell lung cancer
PBMC: Peripheral blood mononuclear cell
ABZI: Amidobenzimidazole
NK: Natural killer
5-FU: 5-fluorouracil
HNSCC: Head neck squamous cell carcinoma
ALT: Alternative lengthening of telomeres
DMBA: 7,12-dimethylbenz(a)anthracene
TSCC: Tongue squamous cell carcinoma
MDSC: Myeloid-derived suppressor cell
IDO: Indoleamine 2,3-dioxygenase
LLC: Lewis lung carcinoma
CX43: Connexin 43
PCDH7: Protocadherin 7
MSC: Mesenchymal stromal cell
